# Common Variants in Promoter of *ADTRP* Associate with Early-Onset Coronary Artery Disease in a Southern Han Chinese Population

**DOI:** 10.1371/journal.pone.0137547

**Published:** 2015-09-16

**Authors:** Er-Wen Huang, Long-Yun Peng, Jin-Xiang Zheng, Dan Wang, Qu-Yi Xu, Lei Huang, Qiu-Ping Wu, Shuang-Bo Tang, Bin Luo, Shui-Ping Liu, Xiao-Shan Liu, Zhao-Hui Li, Li Quan, Yue Li, He Shi, Guo-Li Lv, Jian Zhao, Jian-Ding Cheng, Chao Liu

**Affiliations:** 1 Department of Forensic Pathology, Zhongshan School of Medicine, Sun Yat-Sen University, Guangzhou, Guangdong, China; 2 Guangzhou Forensic Science Institute, Guangzhou, Guangdong, China; 3 Department of Cardiology, The First Affiliated Hospital, Sun Yat-Sen University, Guangzhou, Guangdong, China; 4 Key Laboratory of Molecular Biophysics of the Ministry of Education, Cardio-X Institute, College of Life Science and Technology and Center for Human Genome Research, Huazhong University of Science and Technology, Wuhan, Hubei, China; Medical University Hamburg, University Heart Center, GERMANY

## Abstract

The first genome-wide association study for coronary artery disease (CAD) in the Han Chinese population, we reported recently, had identified rs6903956 in gene *ADTRP* on chromosome 6p24.1 as a novel susceptibility locus for CAD. The risk allele of rs6903956 was associated with decreased mRNA expression of *ADTRP*. To further study the correlation of *ADTRP* expression and CAD, in this study we evaluated the associations of eight common variants in the expression-regulating regions of *ADTRP* with CAD in the Southern Han Chinese population. Rs169790 in 3’UTR, rs2076189 in 5’UTR, four SNPs (rs2076188, rs7753407, rs11966356 and rs1018383) in promoter, and two SNPs (rs3734273, rs80355771) in the last intron of *ADTRP* were genotyped in 1716 CAD patients and 1572 controls. The correlations between these loci and total or early-onset CAD were investigated. None of these loci was discovered to associate with total CAD (*P* > 0.05). However, with early-onset CAD, significant both allelic and genotypic associations of rs7753407, rs11966356 and rs1018383 were identified, after adjustment for risk factors of age, gender, hypertension, diabetes, lipid profiles and smoking (adjusted *P* < 0.05). A haplotype AGCG (constructed by rs2076188, rs7753407, rs11966356 and rs1018383) was identified to protect subjects from early-onset CAD (OR = 0.332, 95% CI = 0.105–0.879, adjusted *P* = 0.010). Real-time quantitative reverse transcription polymerase chain reaction assay showed that the risk alleles of the associated loci were significantly associated with decreased expression of *ADTRP* mRNA. Moreover, the average level of *ADTRP* mRNA expression in early-onset CAD cases was significantly lower than that in controls. Our results provide new evidence supporting the association of *ADTRP* with the pathogenesis of early-onset CAD.

## Introduction

Coronary artery disease (CAD) is one of the leading causes of morbidity and mortality worldwide [1, 2]. It is a complex disease caused by multiple environmental and genetic factors and interactions between them [3, 4]. Approximately 50% of CAD can be explained by heritable variants. Over the past decade, linkage analysis and candidate gene association studies have identified several genes involved in the pathogenesis of CAD [5–7]. Genome-wide association studies (GWAS) have identified dozens of susceptibility loci for CAD in western populations in the past few years [8–12].

Recently we reported the first GWAS for CAD in the Han Chinese population, in which the subjects were from Beijing, Hubei, Shandong and Northern China [13]. In that study, rs6903956 within intron 1 of gene *ADTRP* on chromosome 6p24.1 was identified to be significantly associated with susceptibility to CAD. Moreover, the risk allele A of rs6903956 was discovered to associate with decreased *ADTRP* mRNA expression, suggesting that low levels of *ADTRP* expression may be associated with CAD. However, stronger evidence about the involvement of *ADTRP* in the pathogenesis of CAD remains to be presented.

To gain further evidence about the association between *ADTRP* and CAD, and to verify the link of *ADTRP* expression level and CAD, we carried out another case-control study for CAD in the Han Chinese population from Guangdong—a subpopulation of the Southern Han Chinese population (CHS). Known SNPs in expression-regulating regions of *ADTRP* were interrogated. Sequence within 5kb upstream of the transcription start site (TSS) was considered to include almost the whole promoter region. All SNPs in the 5kb promoter-including region and 5’UTR of *ADTRP* were genotyped or not based on three criteria: 1) it has two alleles in each population in 1000GENOMES and HapMap databases, 2) it has a MAF > 0.1 in each population, 3) only one of the loci that have strong Linkage disequilibrium (LD) with each other in each population was genotyped. Consequently, four SNPs (rs2076188, rs7753407, rs11966356 and rs1018383, respectively 96bp, 938bp, 3844bp, 4922bp upstream of the TSS) in the promoter, one SNP (rs2076189) in the 5’UTR of *ADTRP* were selected. No SNP meets the above mentioned criteria in the 3’UTR of *ADTRP*. Therein rs169790 has the highest MAF in the Southern Han Chinese (CHS) of 1000GENOMES and the Han Chinese in Beijing (HCB) of HapMap databases (MAF = 0.045 in CHS, 0.056 in HCB). To avoid missing the information about the association between the 3’UTR of *ADTRP* and CAD, we selected rs169790 for test. Intronic variants, especially those near the promoter or 3’UTR regions, were usually demonstrated to be associated with phenotypes [13, 14]. Loci within 1kb upstream of the last exon were taken into consideration. In this region only rs3734273 meets the above criteria. Rs80355771 is a unique SNP that has a MAF > 0.4 in CHS and CHB in 1000GENOMES database, moreover, no LD information between the two loci was available in CHS or CHB. Therefore, both rs3734273 and rs80355771 were also selected. Associations of the aforementioned eight SNPs (four in the promoter, one in the 5’UTR, one in the 3’UTR and two in the last intron of *ADTRP*) with CAD were investigated.

## Methods

### Study subjects

All subjects were clinically examined at the Department of Cardiology, the First Affiliated Hospital of Sun Yat-Sen University (Guangzhou, China), and were of self-described Han ethnic origin and from south China-located Guangdong province. All the subjects participated in this study with written consents. This study was approved by the ethics committee of Sun Yat-Sen University and complied with the Declaration of Helsinki.

Eligible standards of CAD conform to the diagnostic guideline declared by American Heart Association: 1) ≥ 70% luminal stenosis in not less than one main coronary arteries diagnosed by coronary angiography (CAG), or 2) percutaneous coronary intervention, or 3) coronary artery bypass graft, or 4) myocardial infarction (MI), or 5) 50% ≤ luminal stenosis < 70% and simultaneously younger than 50 (male)/55 (female) years old, or 6) 25% ≤ luminal stenosis < 50% and younger than 40 (male)/45 (female) years old. The diagnostic criteria of MI was 1) with classic chest pain lasting longer than 30 minutes, 2) characteristically ischemic electrocardiographic patterns and 3) elevation of cardiac enzymes. CAD patients firstly diagnosed not older than 50 (male)/55 (female) years were classified as early-onset CAD; otherwise, they were classified as late-onset CAD [11]. Subjects with congenital heart disease, childhood hypertension, typeIdiabetes, myocardial bridge or myocardial spasms were excluded.

Control subjects include unrelated individuals 1) who were verified by CAG to be free of coronary artery stenosis and not younger than 45 (male)/50 (female) years, or 2) who were verified by electrocardiography to be free of myocardial ischemia, not younger than 40 (male)/45 (female) years and without history of CAD/MI symptoms.

Other clinic data collected included gender, age, total cholesterol, triglyceride, low-density lipoprotein cholesterol (LDL-c), high-density lipoprotein cholesterol (HDL-c), diabetes, hypertension, and history of smoking. Diabetes was diagnosed as a fasting plasma glucose concentration ≥ 7.0 mmol/L. Hypertension was defined as systolic blood pressure ≥ 140 mm Hg/diastolic blood pressure ≥ 90 mm Hg. We had not gotten enough detailed information about the medications for lipids, diabetes, and hypertension of most enrolled subjects. Thus, to eliminate the influences of the medications on the results of association analysis, only subjects who did not take medications for lipids, diabetes and hypertension were included. Consequently, 1716 CAD patients and 1572 non-CAD controls were enrolled in this study.

### DNA preparation and SNP genotyping

Genomic DNA was extracted from peripheral blood using Wizard^®^ Genomic DNA Purification Kit (Promega, Madison, WI, USA) according to the manufacturer’s protocol.

SNPs were genotyped using a MassARRAY^®^ Genetic Analysis System (Sequenom, San Diego, California, USA). Primers used for genotyping were designed by AssayDesigner3.1. Polymerase chain reaction (PCR) volume in MassArray system contained 0.5 μL of 10 x PCR buffer, 0.4 μL of 25 mM MgCl_2_, 0.1 μL of 25 mM dNTP Mix, 1 μL of 0.5 μM primer Mix, 0.2 μL of 5 U/μL HotStar Taq enzyme, 1 μL of 10 ng/μL DNA template and 1.8 μL of water. SAP reaction volume contained 0.17 μL of SAP buffer, 0.3 μL of 1.7 U/μL SAP enzyme, 1.53 μL of nanopure water. Components and reagents of extension reaction included 0.2 μL of iPLEX Buffer Plus, 0.2 μL of iPLEX Termination Mix, 0.94 μL of iPLEX Extend Primer Mix, 0.041 μL of iPLEX Enzyme, 0.619 μL of nanopure water, 7 μL of PCR + SAP product. 20 samples were randomly selected for direct DNA sequencing to verify the genotyping results. All sequencing results matched that by the MassArray method.

### Real-time quantitative reverse transcription PCR (qRT-PCR)

RNA was extracted from peripheral blood cells using TRIzol reagent (Invitrogen) according to the manufacturer’s instructions. Real-time qRT-PCR was performed using OneStep RT-PCR Kit (Qiagen) with GC Green. *ADTRP* cDNA was amplified with forward primer 5'-CCT GGA TGA TGT GCT GAA AAG AAC-3' and reverse primer 5'-AGG ATC CAG AAT GCC AAA AAT ACA-3', as described in the previous study [13]. *ACTB* was used as an internal control and amplified with forward primer 5'-GGA CTT CGA GCA GGA GAT GG-3' and reverse primer 5'-GCA CCG TGT TGG CGT AGA GG-3' [13]. Each reaction was carried out in triplicate as described previously [13].

### Statistic analysis

Statistics Package for Social Sciences software (SPSS, version 13.0) was used for statistic analysis. Deviation of genotype frequencies from Hardy-Weinberg equilibrium (HWE) was estimated using the χ^2^ test. Allelic and genotypic association of single SNP with CAD was assessed using Pearson’s χ^2^ test or Fisher exact test. Odds ratio (OR) and 95% confidence interval (CI) were estimated using the χ^2^ test. Age, gender, lipid profiles, hypertension, diabetes, smoking history were incorporated as covariates by multivariable logistic regression analysis. LD was analyzed by HaploView (version 4.2). Associations between haplotypes and CAD were estimated by Pearson’s χ^2^ test. For mRNA expression comparison, unpaired 2-tailed Student’s t test was used. Statistical power was calculated with Quanto system.

## Results

### Characteristics of the study cohort

A CHS cohort including 1716 CAD patients and 1572 controls was enrolled in this case-control association study. CAD-related clinical characteristics of the population are summarized in Table 1. The ratio of males in cases was significantly higher than that in controls (*P* = 9.0×10^−4^, Table 1). Hypertension, type 2 diabetes and smoking were more prevalent in patients than in controls (*P* < 0.05, Table 1). In patients, the levels of triglyceride and LDL-c were higher while HDL-c level was lower than in controls (*P* < 0.05, Table 1). There was no significant difference in total cholesterol level between two groups (*P* = 0.061, Table 1).

**Table 1:** Clinical and biochemical characteristics of the study subjects.

Characteristics	Cases (n = 1716)	Controls (n = 1572)	*P*
Gender (male/female)	1192/524	805/767	0.0009
Age ^a^ (years)	64.86 ± 11.422	59.70 ± 12.106	0.0017
Hypertension (n/%)	1173/68.4%	854/54.3%	0.013
T2DM (n/%)	378/22.0%	162/10.3%	0.0045
Tch (mmol/L)	4.35 ± 1.110	4.41 ± 1.196	0.061
TG (mmol/L)	1.470 (0.866, 1.955)	1.183 (0.800, 1.564)	0.0028
LDL-C (mmol/L)	2.84 (1.98, 3.29)	2.61 (2.05, 3.12)	0.022
HDL-C (mmol/L)	1.03 ± 0.325	1.24± 0.401	0.0004
Smoker (n/%)	246/14.3%	132/8.4%	0.03

^a^Age at the first diagnosis for CAD and age at the time of enrollment for control. T2DM, type 2 diabetes mellitus; Tch, total cholesterol; TG, triglyceride; LDL-C, low-density lipoprotein cholesterol; HDL-C, high-density lipoprotein cholesterol.

### Genotyping results

HWE test revealed that genotype frequencies of each SNP did not deviate significantly from HWE expectations in total population (*P*_HWE_ > 0.05, Table 2).

**Table 2:** Analysis of allelic association of eight SNPs with CAD.

SNP	MAF	Risk allele frequency (case/control)	*P*_HWE_	*P*_obs_	OR_obs_ (95% CI)	*P*_adj_	OR_adj_ (95% CI)
For total CAD							
rs1018383	0.419 (G)	A (0.60/0.56)	0.643	0.312	1.111 (0.625, 1.713)	0.202	1.318 (0.796, 2.317)
rs11966356	0.408 (C)	T (0.60/0.56)	0.614	0.299	1.044 (0.765, 1.754)	0.125	1.220 (0.738, 2.070)
rs7753407	0.421 (G)	A (0.60/0.56)	0.606	0.287	1.132 (0.778, 1.644)	0.113	1.185 (0.701, 1.845)
rs2076188	0.423 (G)	A (0.61/0.59)	0.088	0.125	1.236 (0.909, 1.677)	0.674	1.128 (0.700, 1.999)
rs2076189	0.256 (A)	A (0.23/0.21)	0.301	0.263	1.157 (0.820, 1.704)	0.558	1.409 (0.782, 1.785)
rs80355771	0.480 (DEL)	A (0.53/0.51)	0.429	0.496	1.088 (0.740, 1.565)	0.606	1.263 (0.624, 1.332)
rs3734273	0.235 (T)	C (0.79/0.73)	0.666	0.194	1.354 (0.855, 2.941)	0.524	1.113 (0.786, 1.921)
rs169790	0.076 (A)	A (0.07/0.06)	0.535	0.526	1.109 (0.604, 3.188)	0.465	1.274 (0.554, 3.607)
For early-onset CAD							
rs1018383	0.401 (G)	A (0.625/0.566)	0.313	0.171	1.886 (0.883, 2.854)	0.007	3.357 (1.162, 7.977)
rs11966356	0.401 (C)	T (0.625/0.566)	0.309	0.166	1.812 (0.851, 2.699)	0.007	3.219 (1.145, 7.732)
rs7753407	0.408 (G)	A (0.625/0.549)	0.333	0.175	1.759 (0.848, 2.698)	0.006	3.205 (1.137, 7.682)
rs2076188	0.399 (G)	A (0.650/0.551)	0.108	0.204	1.616 (0.814, 2.559)	0.094	1.213 (0.894, 2.596)
rs2076189	0.225 (A)	A (0.258/0.192)	0.510	0.218	1.845 (0.800, 2.963)	0.398	2.055 (0.599, 3.125)
rs80355771	0.417 (DEL)	A (0.638/0.533)	0.644	0.143	1.471 (0.813, 3.124)	0.514	1.714 (0.627, 6.638)
rs3734273	0.221 (T)	C (0.811/0.747)	0.497	0.297	1.562 (0.714, 4.033)	0.333	2.182 (0.628, 7.563)
rs169790	0.068 (A)	A (0.070/0.054)	0.286	0.662	1.387 (0.400, 5.797)	0.367	3.561 (0.315, 29.436)

MAF, minor allele frequency; *P*_HWE_, *P* values for Hardy-Weinberg equilibrium tests; *P*_obs_, observed *P* value; *P*_adj_, *P* value after adjustment for covariates of gender, age, hypertension, diabetes, blood lipid profiles, smoking; OR_obs_, observed odds ratio; OR_adj_, odds ratio after adjustment for the covariates; CI, confidence interval.

In total CAD, none of the eight SNPs had a significantly different allele frequency, compared to controls, no matter whether adjustment were made for the covariates of gender, age, hypertension, type 2 diabetes, lipid profiles and smoking status (*P*_obs_ > 0.05, *P*_adj_ > 0.05, Table 2). Similarly, no significant genotypic association was discovered between each SNP and total CAD (*P*_obs_ > 0.05, *P*_adj_ > 0.05, Table 3).

**Table 3:** Analysis of genotypic association of eight SNPs with CAD.

SNP	Model	*P*_obs_	*P*_adj_	OR_adj_ (95% CI)
For total CAD				
rs1018383	Dominant	0.562	0.634	0.999 (0.526, 2.208)
	Recessive	0.811	0.753	1.121 (0.595, 1.963)
	Additive	0.647	0.706	1.077 (0.672, 1.858)
rs11966356	Dominant	0.605	0.852	1.143 (0.564, 2.886)
	Recessive	0.534	0.737	1.064 (0.789, 2.492)
	Additive	0.713	0.622	1.156 (0.637, 1.924)
rs7753407	Dominant	0.475	0.573	1.201 (0.523, 2.877)
	Recessive	0.239	0.325	1.111 (0.764, 2.246)
	Additive	0.356	0.512	1.201 (0.705, 1.884)
rs2076188	Dominant	0.107	0.074	1.798 (0.992, 2.573)
	Recessive	0.343	0.620	1.053 (0.666, 1.879)
	Additive	0.268	0.129	1.332 (0.938, 1.752)
rs2076189	Dominant	0.744	0.521	1.134 (0.652, 1.964)
	Recessive	0.292	0.107	2.037 (0.815, 10.001)
	Additive	0.138	0.256	1.174 (0.885, 2.362)
rs80355771	Dominant	0.434	0.142	0.558 (0.353, 1.476)
	Recessive	0.351	0.622	1.084 (0.604, 1.846)
	Additive	0.624	0.508	0.891 (0.622, 1.534)
rs3734273	Dominant	0.808	0.413	0.723 (0.335, 1.687)
	Recessive	0.265	0.234	1.301 (0.813, 2.410)
	Additive	0.157	0.272	1.109 (0.681, 1.752)
rs169790	Dominant	0.469	0.701	1.167 (0.536, 2.449)
	Recessive	1.000	1.000	/
	Additive	0.514	0.326	1.448 (0.734, 2.068)
For early-onset CAD				
rs1018383	Dominant	0.333	0.529	1.674 (0.315, 8.534)
	Recessive	0.210	0.002	8.737 (1.968, 50.016)
	Additive	0.378	0.030	3.561 (1.095, 8.753)
rs11966356	Dominant	0.334	0.526	1.665 (0.313, 8.522)
	Recessive	0.217	0.002	8.741 (1.977, 50.016)
	Additive	0.372	0.028	3.568 (1.099, 8.845)
rs7753407	Dominant	0.331	0.520	1.659 (0.309, 8.510)
	Recessive	0.221	0.002	8.759 (1.982, 50.214)
	Additive	0.366	0.028	3.564 (1.087, 8.828)
rs2076188	Dominant	0.405	0.643	1.711 (0.389, 7.726)
	Recessive	0.710	0.066	5.138 (0.976, 24.372)
	Additive	0.195	0.107	2.064 (0.953, 2.846)
rs2076189	Dominant	0.168	0.315	1.137 (0.598, 3.472)
	Recessive	0.828	0.235	7.657 (0.521, 96.378)
	Additive	0.211	0.269	1.652 (0.554, 4.834)
rs80355771	Dominant	0.537	0.416	2.255 (0.396, 12.412)
	Recessive	0.198	0.256	1.513 (0.378, 7.025)
	Additive	0.356	0.454	1.107 (0.555, 3.286)
rs3734273	Dominant	0.722	0.287	6.431 (0.746, 15.190)
	Recessive	0.268	0.314	1.685 (0.419, 7.883)
	Additive	0.479	0.262	2.666 (0.718, 5.407)
rs169790	Dominant	0.403	0.236	4.184 (0.369, 17.533)
	Recessive	/	/	/
	Additive	0.746	0.414	1.951 (0.623, 6.885)

*P*_obs_, observed *P* value; *P*_adj_, *P* value after adjusted for covariates of gender, age, hypertension, diabetes, blood lipid profiles, smoking; OR_adj_, odds ratio after adjustment for the covariates; CI, confidence interval.

When only early-onset CAD was analyzed, neither allelic nor genotypic association of any locus was observed (*P*_obs_ > 0.05, Tables 2 and 3). However, after the adjustment, significance for both allelic and genotypic associations of three promoter-locating SNPs, rs7753407, rs11966356 and rs1018383, were achieved. Allele A of rs7753407 had an adjusted OR of 3.205 in early-onset CAD compared to controls (95% CI = 1.137–7.682, *P*_adj_ = 6.0×10^−3^, Table 2). Allele T of rs11966356 had an adjusted OR of 3.219 (95% CI = 1.145–7.732, *P*_adj_ = 7.0×10^−3^, Table 2). Similarly, allele A of rs1018383 was significantly more frequent in early-onset CAD than in control group (*P*_adj_ = 7.0×10^−3^, OR_adj_ = 3.357, 95% CI = 1.162–7.977, Table 2). Rs7753407 was significantly associated with early-onset CAD assuming either a recessive or additive model (*P*_adj_ = 0.002 and 0.028, respectively, Table 3). Similar results were discovered for rs11966356 (*P*_adj_ = 0.002 and 0.028, respectively, Table 3) and rs1018383 (*P*_adj_ = 0.002 and 0.030, respectively, Table 3). Neither allelic nor genotypic adjusted association was significant of rs2076188, rs2076189, rs80355771, rs3734273, rs169790 with early-onset CAD (*P*_adj_ > 0.05, Tables 2 and 3).

**Fig 1:**
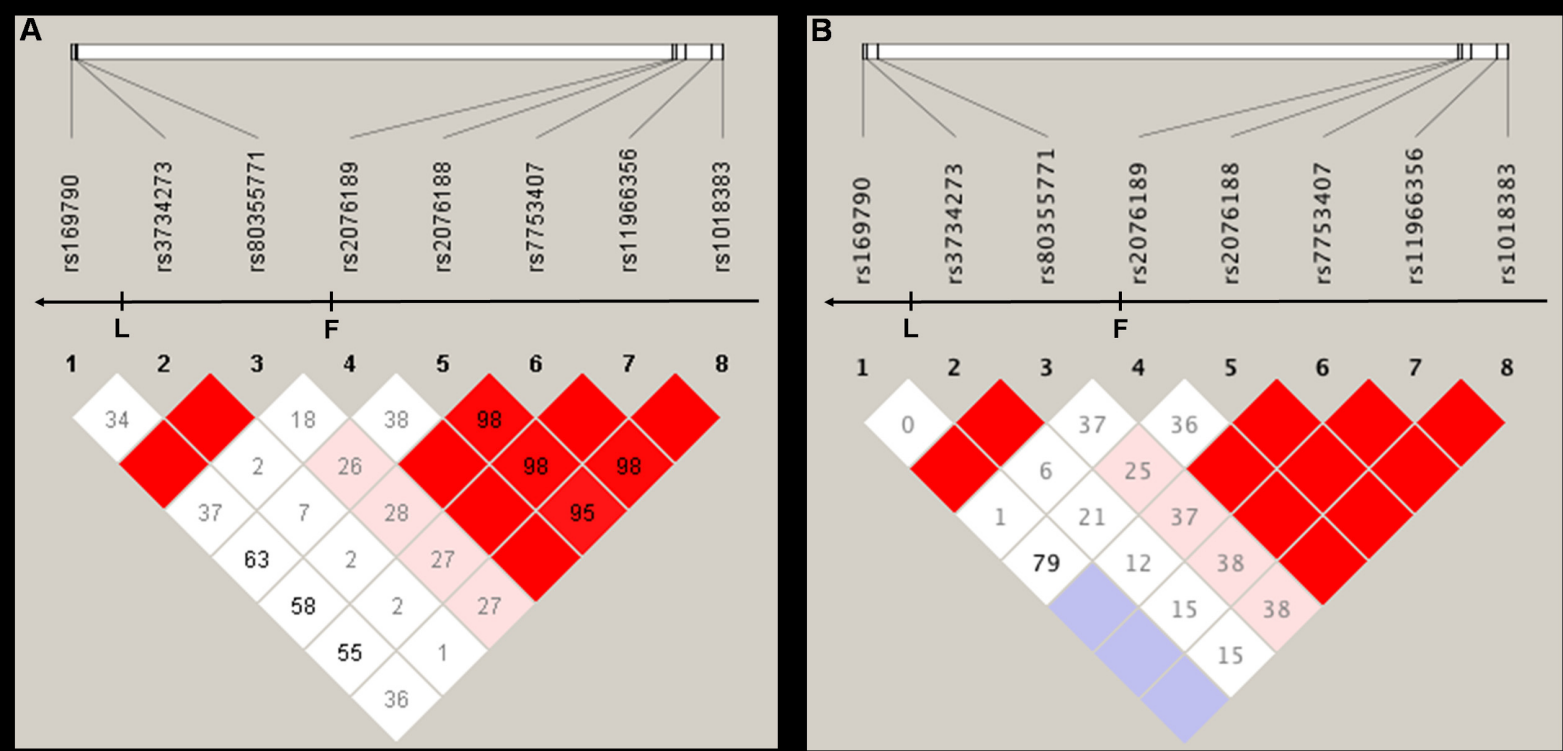
Overview of LD structure among eight SNPs. A was constructed based on the genotype data of all the subjects in this work, B was constructed based on the genotype data of subjects excluding late-onset CAD patients. D’ was signed with a number in a diamond. Red diamond without a number refers to D’ = 100. F refers to exon 1, L refers to the last exon. Arrow refers to the transcription direction of *ADTRP*.

We constructed a LD map for all the tested SNPs based on the genotyping data. Strong linkages between rs80355771 and rs3734273 (block 1, Table 4, Fig 1A and 1B),among rs2076188, rs7753407, rs11966356 and rs1018383 (block 2, Table 4, Fig 1A and 1B) were observed in either total or early-onset CAD analysis. Three main haplotypes CA, CDEL., TDEL. in block 1, and four main haplotypes GATA, AGCG, AATA, AGTA in block 2 were observed (Table 4). We discovered no significant association between any haplotype and total CAD (*P*_obs_ > 0.05, *P*_adj_ > 0.05, Table 4). However, significant association was identified between haplotype AGCG and early-onset CAD (*P*_adj_ = 0.010, OR_adj_ = 0.332, 95% CI = 0.105–0.879, Table 4), indicating that AGCG was a protective haplotype for early-onset CAD.

**Table 4:** Analysis of haplotypic association of eight SNPs with CAD.

Block	Haplotype	Frequency	Frequencies in Case, Control	*P*_obs_	*P*_adj_	OR_adj_ (95% CI)
For total CAD						
Block 1	CA	0.546	0.530, 0.554	0.643	0.479	0.912 (0.663, 2.453)
	CDEL.	0.265	0.317, 0.241	0.258	0.316	1.878 (0.812, 4.337)
	TDEL.	0.188	0.152, 0.204	0.496	/	1.000
Block 2	GATA	0.420	0.411, 0.426	0.547	0.623	0.764 (0.256, 4.686)
	AGCG	0.413	0.395, 0.422	0.189	0.245	0.801 (0.469, 3.180)
	AATA	0.155	0.186, 0.135	0.373	0.571	1.542 (0.384, 3.855)
	AGTA	0.011	0.007, 0.016	0.604	/	1.000
For early-onset CAD						
Block 1	CA	0.552	0.614, 0.523	0.268	0.566	1.293 (0.513, 5.084)
	TDEL.	0.228	0.175, 0.242	0.518	0.376	0.560 (0.389, 2.607)
	CDEL.	0.220	0.211, 0.235	0.682	/	1.000
Block 2	GATA	0.413	0.406, 0.421	0.915	0.453	0.507 (0.418, 2.724)
	AGCG	0.409	0.373, 0.434	0.361	0.010	0.332 (0.105, 0.879)
	AGTA	0.010	0.005, 0.013	0.576	0.999	/
	AATA	0.169	0.216, 0.132	0.163	/	1.000

*P*_obs_, observed *P* value; *P*_adj_, *P* value after adjusted for gender, age, hypertension, diabetes, blood lipid profiles, smoking history; OR_adj_, odds ratio after adjustment for the covariates; CI, confidence interval. Block 1, constructed with rs3734273 and rs80355771. Block 2, constructed with rs2076188, rs7753407, rs11966356, rs1018383.

**Fig 2:**
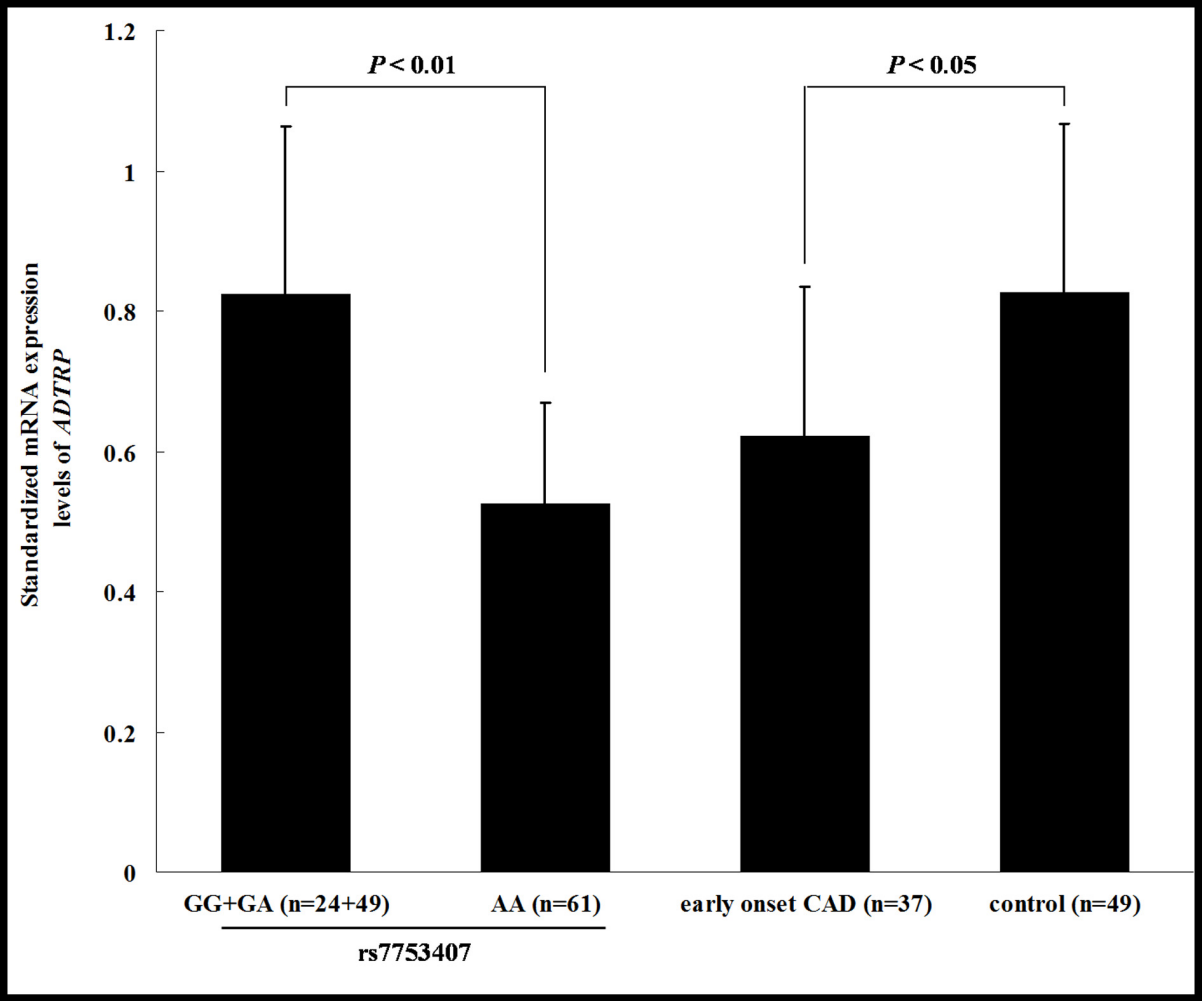
Difference of *ADTRP* mRNA expression between groups with different genotypes of rs7753407, and between early-onset CAD cases and controls. Levels of *ADTRP* mRNA expression in leukocytes from 134 randomly selected subjects were measured using real-time qRT-PCR. The average level of it in 61 subjects with the risk genotype AA of rs7753407 was significantly lower than that in 73 subjects with genotype GG or GA (*P* < 0.01, bars represent mean±S.E.). The average level in 37 early-onset CAD cases was significantly lower than that in 49 controls (*P* < 0.05, bars represent mean±S.E.).

We further tested the effect of the associated SNPs on the expression of *ADTRP* mRNA. The mRNA expression level of *ADTRP* in leukocytes from 134 randomly selected subjects was measured using real-time qRT-PCR. As shown in Fig 2, the average expression level of *ADTRP* mRNA in 61 individuals with the risk genotype AA of rs7753407 was significantly lower than in 73 individuals with genotype GG or GA (*P* < 0.01). Similar results of rs11966356 and rs1018383 were obtained (data not shown). 37 early-onset CAD cases and 49 controls were included in the 134 individuals. We further obtained the result that the average level of *ADTRP* mRNA expression in early-onset CAD cases was significantly lower than that in controls (*P* < 0.05, Fig 2). These results suggested that decreased expression of *ADTRP* is a risk factor for CAD, in consistent with the result of the previous study [13].

## Discussion

As we recently reported, a GWAS has revealed that rs6903956 in intron 1 of *ADTRP* on chromosome 6p24.1 was associated with CAD in a Han Chinese population [13]. The association was later replicated respectively in a Han Chinese cohort [15] and a Chinese ethnic Singaporean cohort [16]. Rs6903956 was also demonstrated to associate with the mRNA expression of *ADTRP* [13].

Because mRNA expression is regulated by promoter, 5’UTR and 3’UTR regions, in this study we selected several SNPs in these regulatory regions, to study the associations of them with CAD in CHS.

Herein, three promoter-locating SNPs (rs7753407, rs11966356, rs1018383) were identified as susceptibility loci for early-onset CAD. Significance for both allelic and genotypic associations between the three SNPs and early-onset CAD were discovered. Moreover, a haplotype AGCG (constructed with rs2076188, rs7753407, rs11966356, rs1018383) was also significantly associated with early-onset CAD. We also analyzed the associations between these SNPs and late-onset CAD, but no positive result was discovered (data not shown). We speculated that such a difference between early and late-onset (or total) CAD resulted from the difference in the contribution of genetic factor to early and late-onset CAD.

Bioinformatic analysis showed that neither of the promoter-locating SNPs studied here was on any transcription factor-binding site. However, based on the 1000GENOMES database, we predicted that two other variants, rs2056952 and rs6913633, were strongly linked to LD block 2 (*D*’ = 1, r^2^ = 1 for rs2056952 with rs7753407, *D*’ = 0.979, r^2^ = 0.901 for rs6913633 with rs7753407, http://www.ensembl.org/index.html, 1000GENOMES:phase_1_CHS). Moreover, the two variants are respectively on the binding sites of transcription factors Nkx-2 and USF (predicted score were 94.1 and 93.0 respectively, http://www.cbrc.jp/research/db/TFSEARCH.html). Interestingly, USF has been reported to be involved in insulin-triggered promotion of lipogenesis and increase of triglyceride levels [17, 18], which is an independent risk factor for CAD. Nkx-2 has been revealed to play roles in regulating β-cell function and insulin secretion [19–21]. These information together suggest that ADTRP may be involved in insulin-mediated metabolism regulation and metabolic disorders, such as CAD. Androgen is a protective factor for atherosclerosis [22–25]. And it was demonstrated that androgen-regulated expression and activation of Tissue Factor Pathway Inhibitor, which functions in the incipient point of thrombosis and atherosclerosis—vascular endothelial cell dysfunction, was ADTRP-dependent [26]. Moreover, ADTRP was recently found to be up-regulated by Peroxisome Proliferator-Activated Receptor γ in human macrophages and atherosclerosis lesions [27]. These reports seem to correlate ADTRP more directly with CAD.

Soon after the GWAS firstly identified rs6903956 in *ADTRP* as a susceptibility locus in Han Chinese, another GWAS in the same ethnic population failed to verify whether this gene is associated with CAD [28]. One possible explanation for this inconsistence is the difference between the two studies in population stratification. Another explanation may be the difference in using gene chips which had different genome coverage density. Shortly before we submitted this manuscript, rs6903956 was revealed to associate with CAD in a Japanese cohort, but contradictorily, the minor allele A of rs6903956 was a protective allele [29], rather than a risk one as the former studies reported. Such a contradiction may be attributed to differences in genetic structures and interactions with environmental factors, as the authors speculated. Due to weak LD between rs6903956 and the SNPs genotyped in this work, we could not eliminate the conflict in the present study.

In conclusion, we presented additional evidence that supports the association of *ADTRP* with CAD. Our results indicated that expression levels of *ADTRP* regulated by the promoter may be involved in the pathogenesis of early-onset CAD. However, further studies, especially functional studies, are needed to fully confirm the role of *ADTRP* in the pathogenesis of CAD.
